# Dissociating rehearsal and refreshing in the maintenance of verbal information in 8-year-old children

**DOI:** 10.3389/fpsyg.2015.00011

**Published:** 2015-01-23

**Authors:** Gérome Mora, Valérie Camos

**Affiliations:** ^1^Laboratoire d’Etude de l’Apprentissage et du Développement, Université de Bourgogne – Centre National de la Recherche ScientifiqueDijon, France; ^2^Laboratory of Cognitive Development, Fribourg Center for Cognition and Département de Psychologie, Université de FribourgFribourg, Switzerland

**Keywords:** working memory, maintenance, rehearsal, refreshing, children

## Abstract

Recent models of working memory suggest that two systems are involved in verbal working memory: one is dedicated to the maintenance of phonological representations through verbal rehearsal, while the other would maintain multimodal representations through attentional refreshing (Camos et al., 2009; Baddeley, 2012). Previous studies provided evidence on the existence of these two maintenance systems, on their independence, and how they affect recall performance in adults. However, only one study had already explored the relationships between these two systems in children ( Tam et al., 2010). The aim of the present study was to further examine how the two systems account for working memory performance in children. Eight-year-old children performed complex span tasks in which the availability of either the rehearsal or the refreshing was impeded by a concurrent articulation or an attention-demanding task, respectively. Moreover, the phonological similarity of the memoranda was manipulated. Congruently with studies showing that older children can used these maintenance systems, impeding any of the two systems reduced recall performance. Moreover, the manipulation of the two mechanisms did not interact, as previously observed in adults. This suggests that the two maintenance mechanisms are independent in 8-year-old children as they are in adults. However, the results concerning the phonological similarity effect (PSE) differed from what is observed in adults. Whereas the PSE relies only on the availability of rehearsal in adults, a more complex pattern appeared in children: the concurrent articulation as well as the concurrent task modulated the emergence of the PSE.

## INTRODUCTION

Working memory refers to the processes by which information is maintained in the short term while concurrent task is performed. In the past 40 years, the concept of working memory became central in our understanding of human cognition, and it is now recognized as a major predictor of achievement in many cognitive activities (cf. Conway et al., 2007, for review). Among the different processes described as being involved in the maintenance of information, two models of working memory suggest that two specific mechanisms are dedicated to the maintenance of verbal information (Camos et al., 2009; Baddeley, 2012). Subvocal rehearsal and attentional refreshing can be used when verbal material needs to be stored in working memory. Within the framework of one of these models, namely the time-based resource-sharing (TBRS) model, the relationships between the two mechanisms have been recently explored as well as the way their use affects recall performance in adults (Camos and Barrouillet, 2014; Barrouillet and Camos, 2015, for review). The aim of the present study is to extend the examination of the relationships between these two mechanisms to children’s working memory.

Between the two mechanisms in charge of verbal maintenance, subvocal rehearsal is the most studied. It is described as an inner repetition of memoranda using language-based processes akin to those involved in language production, and several well-known effects related to its use have been described in the model of the phonological loop (Baddeley, 1986, 2007). Because rehearsal involves the same processes as language production, a concurrent articulation can impede the functioning of rehearsal (Murray, 1967, 1968; Vallar and Baddeley, 1982; Baddeley et al., 1984). This effect of concurrent articulation has been evidenced through the reduction of memory performance when the possibility to articulate memoranda is suppressed with the verbalisation of non-specific items. A second well-known effect is the word length effect evidenced by a reduction of recall for lists of long words compared to lists of short words. Because lists of short words could benefit from more repetitions than lists of long words in a fixed duration, the former are better maintained in the phonological loop than the latter (Baddeley et al., 1975, 2002; La Pointe and Engle, 1990; Tehan et al., 2001). Finally, storing phonologically similar words leads to more confusion than storing dissimilar words because the phonological loop stores verbal information in a phonological format (Conrad and Hull, 1964; Baddeley, 1966; Lobley et al., 2005). The reduction of recall for similar (vs. dissimilar) words is named the phonological similarity effect (PSE). Aside the phonological loop and its maintenance by subvocal rehearsal, another mechanism, attentional refreshing, has been identified to contribute to the maintenance of verbal information in working memory. Attentional refreshing allows memory traces to be consolidated or reactivated through the recirculation of traces in the focus of attention (Cowan, 1992, 1995; Johnson, 1992; Barrouillet et al., 2004). Recently, the TBRS model suggests that this mechanism is involved in an executive loop in which memory items can be maintained as multiformat representations through refreshing within an episodic buffer (Barrouillet and Camos, 2015). The involvement of such an attentional system in verbal maintenance has been evidenced by the decrease of memory performance when a concurrent task requires attention to be completed, distracting attention from maintenance activities (e.g., Barrouillet et al., 2007, 2011). In a similar vein, the existence of a second system, over and beyond the phonological loop and its rehearsal mechanism, has been recently included in the multi-component model (Baddeley, 2012), although this idea was often mentioned in previous works (e.g., Baddeley and Hitch, 1974; Vallar and Baddeley, 1982; Salamé and Baddeley, 1986; Hitch et al., 1989, 1993; Towse et al., 1998). Thus, two systems, named the phonological and executive loops in the TBRS model, could manage verbal maintenance in working memory.

The existence of two mechanisms in charge of the maintenance of verbal information raises questions about their interrelationships and on how their use may affect recall performance. Hudjetz and Oberauer (2007) provided the first behavioral evidence that refreshing is actually a distinct process from rehearsal. In reading span tasks, these authors manipulated the reading instructions (continuous reading vs. at own pace) in such a way that subvocal rehearsal was more or less impeded. They also varied the pace of presentation of the segments to be read, which affects the availability of attention for maintenance activities. Although recall performance was modulated by the reading instructions and the pace of presentation, the lack of interaction between these two factors contradicts the idea that maintenance relies exclusively on subvocal rehearsal. On the contrary, this suggests that another maintenance mechanism, different from subvocal rehearsal, is implicated in the maintenance of verbal memoranda in working memory. Extended this line of research, Camos et al. (2009) provided further evidence. In a series of experiments, they manipulated independently the availability of rehearsal and refreshing, impeding these mechanisms by introducing a concurrent articulation and increasing concurrent attentional demand, respectively. Whereas the two manipulations resulted in reduced recall performance, they never interacted, resulting in additive effect of rehearsal and refreshing on recall. These authors suggested that the two mechanisms are independent. Their joint use could even enhance working memory performance, with more memory items being recalled when both rehearsal and refreshing can be applied (see also Vergauwe et al., 2014). Because of this independence, the two mechanisms can also be strategically chosen, that is one mechanism can be favored compared to the other depending on instructions or characteristics of the memory task. For example, Camos et al. (2011) showed that young adults favor subvocal rehearsal when they have an attention-demanding task to perform concurrently to the maintenance of verbal items. Rehearsal requiring little attentional resources (Naveh-Benjamin and Jonides, 1984), its use would allow to allocate more attention to the concurrent task. Conversely, adults favor refreshing instead of rehearsal when the memoranda are easily confusable (e.g., lists of phonologically similar words) to reduce the impact of such a confusion on recall. Generalizing this idea, Camos et al. (2013) and Mora and Camos (2013) showed that recall performance in complex span tasks is affected by the characteristics of the memoranda (i.e., phonologically similarity or word length) only when rehearsal is available. When the concurrent task is performed aloud, resulting in a concurrent articulation that impedes rehearsal, both the PSE and the word length effect disappeared. However, such a variation in the occurrence of the phonological similarity and word length effects was independent on the attentional demand induced by the concurrent task. Increased attentional demand reduces recall, but does not affect the phonological similarity and word length effects, bringing a further evidence on the existence of a second attention-based system involved in verbal recall. To summarize, the two maintenance mechanisms described to maintain verbal information in working memory are distinct and independent. Although they can be jointly used, adults can choose to favor one or the other system. However, the use of one or the other mechanism results in different pattern of recall performance. Using subvocal rehearsal makes recall susceptible to the PSE and the word length effect, whereas attentional refreshing depends only on the attentional demand of a concurrent task, which makes attention more or less available for maintenance.

Though the past years allow extensive exploration of the relationships between rehearsal and refreshing in adults, this issue was less studied in children. Nevertheless, many studies have examined the development of each of these maintenance mechanisms independently. Considerable evidence was provided on the emergence of rehearsal at around 7 years of age. At that age, the appearance of lip movements indicates that some verbal repetitions occur (Flavell et al., 1966). The fact that the correlation between speech rate and memory span becomes significant at seven evidences the use of subvocal rehearsal (Gathercole and Adams, 1993; Gathercole et al., 1994). Moreover, recall performance is sensitive to phonological similarity of visually presented memory items in children older than 7, whereas visual similarity affects recall in younger children (e.g., Hitch et al., 1991; Gathercole et al., 1994). However, recent findings questioned this qualitative change showing that children younger than 7 could use rehearsal (Al-Namlah et al., 2006; Tam et al., 2010; Jarrold and Tam, 2011; Henry et al., 2012), or that the PSE is not an adequate index of the emergence of rehearsal (Jarrold and Citroën, 2013). Concerning refreshing, few studies examined it in children (Barrouillet et al., 2009; Camos and Barrouillet, 2011; Gaillard et al., 2011). These previous studies revealed that the reduction of recall performance when the attentional demand of a concurrent task increases occurred in children above 7 years of age, the younger children being insensitive to an increased attention-demand (Barrouillet et al., 2009; Camos and Barrouillet, 2011). Above 7 years of age, the efficiency of refreshing increases to reach a similar level at 14 as in young adults. This improvement in refreshing is a major determinant of the developmental increase in working memory capacity observed in childhood (Gaillard et al., 2011).

Despite the fact that both refreshing and rehearsal would emerge at similar age, only one study examined the relationships between these two maintenance mechanisms in children (Tam et al., 2010). In two experiments involving 6- and 8-year-old children, Tam et al. (2010) varied the opportunities for memory maintenance. Using mostly Brown–Peterson paradigm, they manipulated the type of tasks introduced between the presentation of memory items and their recall. Compared with a simple span condition, the introduction of an unfilled delay allowed examination of the effective use of maintenance mechanisms, as children can freely use rehearsal and refreshing. When a concurrent task was added, the authors introduced either a verbal processing, which impedes rehearsal, or a non-verbal processing to hinder refreshing but to allow rehearsal. In addition, the to-be-memorized words were either phonologically similar or dissimilar. While the phonological similarity of the memoranda was largely tested in immediate serial recall, a very small number of studies examined this effect in working memory tasks, i.e., tasks that require the maintenance of items in face of distracting activities like in a Brown–Peterson or a complex span paradigms (cf. in adults, Tehan et al., 2001; Lobley et al., 2005; Camos et al., 2011, 2013; Macnamara et al., 2011). To our knowledge, Tam et al. (2010) provided in children the first examination of the PSE in a working memory task. Results showed that the unfilled delay led to a reduction of recall performance in both age groups compared to a simple span condition. Moreover, the two types of concurrent task also reduced recall, the verbal task resulting in a greater decrease in recall than the non-verbal task. This detrimental effect of the verbal task was more damaging in 8- than in 6-year-old children. This suggests that 6-year-old children use rehearsal but to a lesser extent than the 8-year olds. In line with this suggestion, the PSE affected recall in both 6- and 8-year-old children. However, this effect disappeared when children performed the concurrent verbal task. This first study brought already a lot of information about the functioning of rehearsal and refreshing in children. The major point raised by the authors was that children younger than 7 showed evidence of use of subvocal rehearsal: a verbal concurrent task impeded their recall that was also sensitive to the phonological similarity of the memoranda. As also observed in adults, the PSE disappeared when a verbal concurrent task was added. Finally, the authors suggested that the stronger effect of the verbal concurrent task could be due to the fact that such a task impairs both rehearsal and refreshing whereas the non-verbal task restraints the use of refreshing only.

Given that only one study examined the relationships between refreshing and rehearsal in children’s working memory, the aim of the present study was to extend this examination. Because our aim was not to debate about the age of emergence of rehearsal use, we focused our investigation on 8-year-old children, for which there is a consensus they are able to use both mechanisms. The procedure used in this study was similar to Camos et al. (2013) and Mora and Camos (2013) studies in adults. The only difference with the previous studies was that memory items were auditory presented to children to assure a verbal encoding of memoranda unbiased by reading proficiency. Thus, this study departed from Tam et al. (2010) in three main aspects. First, in the present study, the opportunity to use rehearsal and refreshing was orthogonally manipulated in a fully crossed design to examine the interactions between the two maintenance mechanisms. As a consequence, children performed four different working memory span tasks in which the use of the two mechanisms was either possible, hindered, or only the use of one of the two mechanisms was impeded. To impede refreshing, a non-verbal task was added concurrently to the maintenance of memory items. This task required to judge the location of series of smileys presented sequentially either in the upper or lower part of a computer screen. To reduce the use of rehearsal, children were asked to repeat “*oui”* (*yes* in French) at a regular pace. This repetition induced an articulation suppression that was independent from the concurrent task children had to perform. This allowed a clear dissociation between the manipulation of the availability of rehearsal and refreshing. Moreover, to control for the amount of repetitions across the different conditions, series of beeps were presented through headphone to help children keeping the pace of repetitions regular. Second, as in Tam et al. (2010), we manipulated the phonological similarity of the memoranda by presenting lists of similar or dissimilar monosyllabic words in Experiment 1. Finally, we used complex span tasks in which the length of memory items was kept constant to extend Tam et al.’s (2010) findings to another working memory paradigm. Moreover, Hitch et al. (1989) found that fixed list lengths were more sensitive to PSE than span procedures. We chose to presented lists of five words for two reasons. First, we wanted to avoid ceiling effect, and some previous studies had already used lists of four items with younger children (e.g., Palmer, 2000; Smith and Jarrold, 2014). Second, previous studies with a spoken presentation in younger children found that shorter lists of three items did not produce PSE, but longer lists of four items did (Hitch et al., 1991; Longoni and Scalisi, 1994). Contrary to Tam et al. (2010) in which the delay of retention varied across list lengths, delays of retention were also kept constant across conditions and experiments in the present study. To summarize, we expected that 8-year-old children should be able to use both rehearsal and refreshing to maintain verbal information in working memory. As a consequence, their recall should be reduced when either rehearsal or refreshing is impeded by concurrent activity such as a concurrent articulation or a concurrent attention-demanding task, respectively. Although rehearsal and refreshing are independent and never interacted in adults’ recall performance, we have no specific prediction concerning children and no previous study examined the interaction between the two maintenance mechanisms in children. From Tam et al. (2010), we can only suspect that impeding the two mechanisms in children should lead to stronger reduction of recall than the impediment of a single mechanism. Finally, Tam et al.’s (2010) previous findings led also to expect that children’s recall should be sensitive to phonological similarity and that this effect should depend on the availability of rehearsal, disappearing under concurrent articulation.

To summarize, the aim of this experiment was to examine the role of rehearsal and refreshing in the maintenance of verbal information in children’s working memory. The material and procedure were an adaptation to children of Camos et al.’s (2013) Experiment 3. Eight-year-old children performed four different complex span tasks in which the availability of rehearsal and refreshing was orthogonally manipulated. Moreover, the to-be-memorized items were presented in lists of phonologically similar or dissimilar words.

## MATERIALS AND METHODS

### PARTICIPANTS

Twenty-three children (10 girls and 13 boys) were recruited from third-year classes across four local primary schools in France. They were all French native speakers, aged between 7 years 11 months and 9 years 2 months, the mean age was 8 years 6 months.

### MATERIAL

Lists of to-be-remembered words were constructed from an initial set of 1143 monosyllabic singular French nouns extracted from *Manu35* sub-database for children in the French *Manulex-Infra* database (Peereman et al., 2007; Ortéga and Lété, 2010). All words were three-phonemes long with a CVC structure. Following Baddeley (1966), 20 lists of five similar words were built with the constraint that words within a list differed on their initial and final phonemes but were similar on their central phoneme (e.g., *poche, robe, mode, coq, bol*). In the database, words with high frequency in Year-3 schoolbooks were preferentially selected. Frequencies were ranged from 5 to 198 per million occurrence (mean = 58, SD = 49). The 20 lists were split in two sets of 10 lists each, in such a way that each list of one set matched a list of the other set on its mean frequency and central phoneme. Lists of dissimilar words were built by mixing words of each set, all words within a dissimilar list differing on their three phonemes. Thus, a set of 10 lists of dissimilar words (D1) was made from the set of 10 lists of similar words (S1), while an other set of 10 lists of dissimilar words (D2) was made from the set of 10 lists of similar words (S2). To avoid that a word was displayed twice along the experiment, half participants was presented with S1 and D2, while the other half was presented with S2 and D1. The lists were recorded in a sound-attenuated booth by a female voice to be auditory presented to children. We verified that the duration of words never exceeded 1000 ms.

### PROCEDURE

**FIGURE 1:**
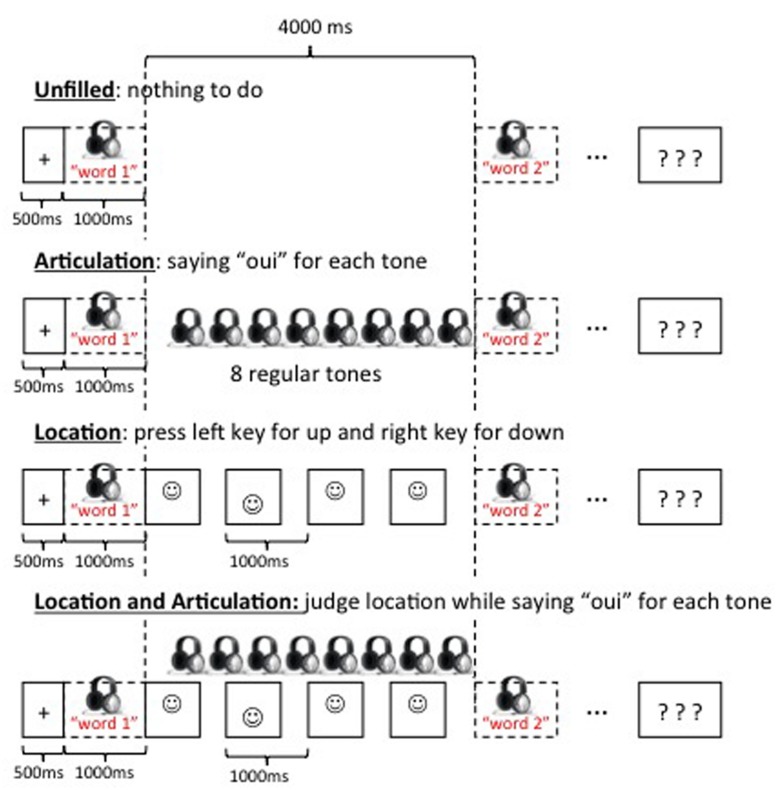
**Illustration of the four complex span tasks**.

The experiment was built with *Psyscope* software (Cohen et al., 1993). Children were seated at about 50 cm from a laptop screen and wore headphone. They performed four different complex span tasks, the order of which was counterbalanced across participants. All the trials in the four span tasks had the same structure (**Figure 1**). A trial started with a fixation cross displayed in the center of the screen for 500 ms. It was followed by a word presented through headphone for 1000 ms. After a 4000-ms delay, the second word appeared for 1000 ms, and so on. When the five words of a list have been presented, each word followed by a 4000-ms delay, participants were cued with a visual signal (i.e., three question marks) to verbally recall words in the same order as they were presented. The children were encouraged to say “don’t know” if they could not remember a word. The experimenter wrote down responses and pressed the space bar to start the next trial when the children were ready. The four complex span tasks differed on the activity performed during the between-word delays. In the *Unfilled* span task, delays remain unfilled, and children did not have any concurrent task to perform. In the *Articulation* span task, children heard series of eight 10-ms tones (32 bits, 44100 Hz) presented every 490 ms through headphone. The first tone appeared 500 ms after the word. Children were instructed to say “*oui*” (“*yes*”) each time they heard a tone to induce a concurrent articulation. In the *Location* span task, children were presented with a series of six 666-ms smileys (2-cm diameter) interleaved with 334-ms white screens. The first smiley appeared in center of the screen immediately after the word. Each smiley was randomly presented in the lower or upper part of the screen, i.e., 1.5-cm apart from the center of the screen. Children were instructed to press as fast as possible the corresponding key, on the right side or on the left side of the keyboard when the square appeared in the lower or upper location, respectively. In the *Articulation and Location* span task, children had to perform simultaneously the concurrent articulation and the location judgment task. Each complex span task began with some practice trials followed by four testing trials. Children received one practice trial in the *Unfilled* span task. They received two practice trials in the *Articulation* and *Location* span tasks to familiarize themselves with the concurrent articulation and the location judgment tasks, and three practice trials in the *Articulation and Location* span task to exercise the combined concurrent articulation and location judgment tasks. For all practice trials, memoranda were forenames to avoid interference with memoranda presented in the testing trials. Response times (RTs) and accuracy were recorded for the location judgment task, and the experimenter counted the number of utterance of “*oui*” during each delay in the tasks with concurrent articulation.

Before performing the four complex span tasks, children did a *Simple* span task, in which five to-be-remembered words were successively displayed, with one word every 1000 ms. The *Simple* span task included one practice trial followed by four testing trials. In the simple and the four complex span tasks, the testing trials included two trials with phonologically similar words and two trials with dissimilar words. Similar and dissimilar lists were randomly assigned to the five tasks and randomly presented in the trials. Words from a given list were also presented in a randomized order, varying across children. The experiment lasted about 30 min.

## RESULTS

The data of four children had to be discarded, because these children were at chance in performing the location judgment task. To analyze performance in the concurrent tasks, analyses of variance (ANOVAs) were conducted with the tasks and the type of lists as within-subject factors on the number of repetitions of “*oui*” for the *Articulation* and *Articulation and Location* span tasks, and on RTs and percentages of correct location judgements for the *Location* and *Articulation and Location* span tasks. The number of repetitions was similar across the lists (7.6, SD = 0.39, for similar, and 7.5, SD = 0.52, for dissimilar lists), *F*(1,18) = 1.57, *p* = 0.23, $\eta_{p}^{2}$ = 0.08. However, less repetitions were produced in the *Articulation and Location* task (6.8, SD = 1.13) than in the *Articulation* span task (8.2, SD = 0.47), *F*(1,18) = 24.02, *p* < 0.0001, $\eta_{p}^{2}$ = 0.57. Although this reduction was relatively small, it could be explained by the difficulty for children to deal with the dual requirement of repetition and location judgment in the *Articulation and Location* task. This effect did not interact with the type of lists, *F*(1,18) = 3.19, *p* = 0.09, $\eta_{p}^{2}$ = 0.15. Concerning accuracy and RTs in the location judgment task, they did not differ across lists (70%, SD = 17, and 501 ms, SD = 77 for the similar, and 70%, SD = 13, and 487 ms, SD = 69, for the dissimilar lists), *F* < 1 for accuracy, and *F*(1,18) = 2.17, *p* = 0.16, $\eta_{p}^{2}$ = 0.11 for RTs. Accuracy was slightly higher in the *Location* (73%, SD = 17) than in the *Articulation and Location* (67%, SD = 12) span tasks, but this effect failed to reach significant, *F*(1,18) = 3.71, *p* = 0.07, $\eta_{p}^{2}$ = 0.17. RTs significantly differed between the two tasks (521 ms, SD = 75 for *Location*, and 468 ms, SD = 62 for *Articulation and Location* span tasks), *F*(1,18) = 8.10, *p* = 0.01, $\eta_{p}^{2}$ = 0.31. The interaction between the type of lists and the tasks was not significant for accuracy and RTs, *F*s < 1.

Concerning recall performance, children experienced difficulties in complying with the instructions. Especially, children did not say “I don’t know” for position in which they forgot the memory word. For example, when recalling three words, a child said “*robe, coq, bol*” without specifying their position in the “*poche, robe, mode, coq, bol*” list. After few unsuccessful reminders, experimenters stopped requiring children to use “I don’t know” for the forgotten words. It was clearly a mistake to use such a recall technique to gain access to order information. This resulted that all children, except three of them, had a null score for recall in correct position in at least one of the 10 experimental conditions (5 tasks × 2 types of lists). As a consequence, to allow statistical analysis, recall performance was scored as the percentage of words recalled regardless position. Besides the fact that a replication using recall in correct position is required, it should be noted that previous findings comforted us on using such a recall score. First, previous studies in children reported an effect of a concurrent task when recall was scored without taken account of order, as it was found in recall in correct position (Camos and Barrouillet, 2011). Second, the PSE affects mostly the item accuracy than the order accuracy [cf. Tehan et al. (2001) and Camos et al. (2013), for working memory tasks; cf. Table 3 in Gupta et al. (2005) for immediate serial recall task]. Finally, because it was important for the purpose of this study to assess the strength of the null hypothesis and as *p*-values do not provide evidence in favor of this hypothesis, we computed the Bayesian Information Criteria (BIC) for each non-significant effect. A probability *pBIC(H0| D)* above 0.50 is conceived as weak evidence, and above 0.75 as positive evidence that the null hypothesis is true (Masson, 2011).

**FIGURE 2:**
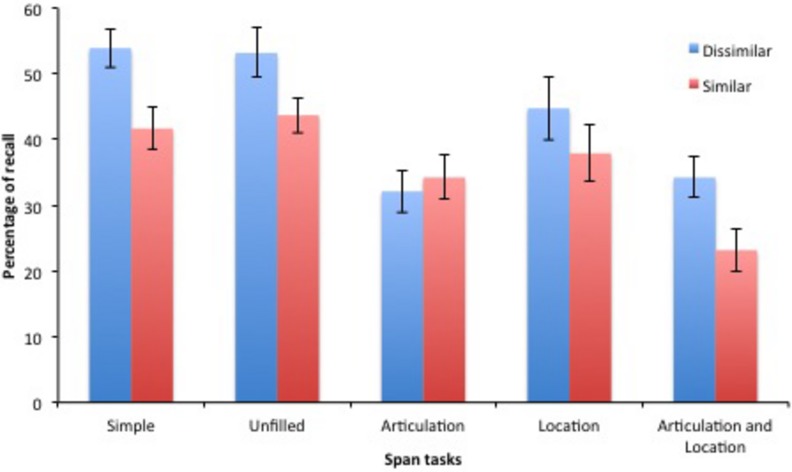
**Percentage of recall according to the type of lists (phonologically dissimilar or similar words), the occurrence of a concurrent articulation and the addition of a concurrent location judgment task.** Y bars represented SE.

The analysis of the *Simple* span task showed that dissimilar lists (54%) were better recalled than similar lists (42%), *F*(1,17) = 8.61, *p* = 0.009, $\eta_{p}^{2}$ = 0.341 (**Figure 2**)^1^. An ANOVA was performed on recall score with the lists (similar vs. dissimilar), the manipulation of a concurrent articulation (silent vs. repetition of “*oui*”) and the introduction of a concurrent task (no task vs. location judgment task) as within-subject factors. This analysis showed that only the three main effects were significant. As in *Simple* span task, phonologically dissimilar lists (41%) were better recalled in complex span tasks than similar (35%) lists, *F*(1,18) = 6.64, *p* = 0.02, $\eta_{p}^{2}$ = 0.27. Introducing a concurrent articulation (31%) strongly reduced recall compare to silent delays of retention (45%), *F*(1,18) = 29.35, *p* < 0.0001, $\eta_{p}^{2}$ = 0.62. Similarly, the addition of a concurrent location judgment task (35% vs. 41% without concurrent task) reduced recall, *F*(1,18) = 9.35, *p* = 0.007, $\eta_{p}^{2}$ = 0.34. More interestingly for the topic of this study, the effect of introducing a concurrent articulation and a concurrent task did not interact, *F* < 1, *pBIC(H0|D)* = 0.79. Moreover, the PSE did not interact with the manipulation of a concurrent articulation, *F* < 1, *pBIC(H0|D)* = 0.73 or of a concurrent location judgment task, *F*(1,18) = 1.05, *p* = 0.32, $\eta_{p}^{2}$ = 0.06, *pBIC(H0|D)* = 0.72. Finally, for the three-way interaction, although it failed to reach significance, *F*(1,18) = 3.11, *p* = 0.10, $\eta_{p}^{2}$ = 0.15, the Bayesian analyses were more in favor of the existence of an interaction, *pBIC(H0| D)* = 0.49. Further analysis showed that the interaction between the PSE and the concurrent task depended on the concurrent articulation. In absence of concurrent articulation, the PSE was significant, *F*(1,18) = 6.77, *p* = 0.02, $\eta_{p}^{2}$ = 0.27, and did not vary across conditions (i.e., *Unfilled* vs. *Location*), *F* < 1, *pBIC(H0| D)* = 0.80. On the contrary, under a concurrent articulation, the PSE interacted with the concurrent task (i.e., *Articulation* vs. *Articulation and Location*), *F*(1,18) = 5.58, *p* = 0.03, $\eta_{p}^{2}$ = 0.24; the PSE being absent in *Articulation*, *F* < 1, *pBIC(H0|D)* = 0.79, but reappearing in *Articulation and Location*, *F*(1,18) = 6.98, *p* = 0.02, $\eta_{p}^{2}$ = 0.28.

**FIGURE 3:**
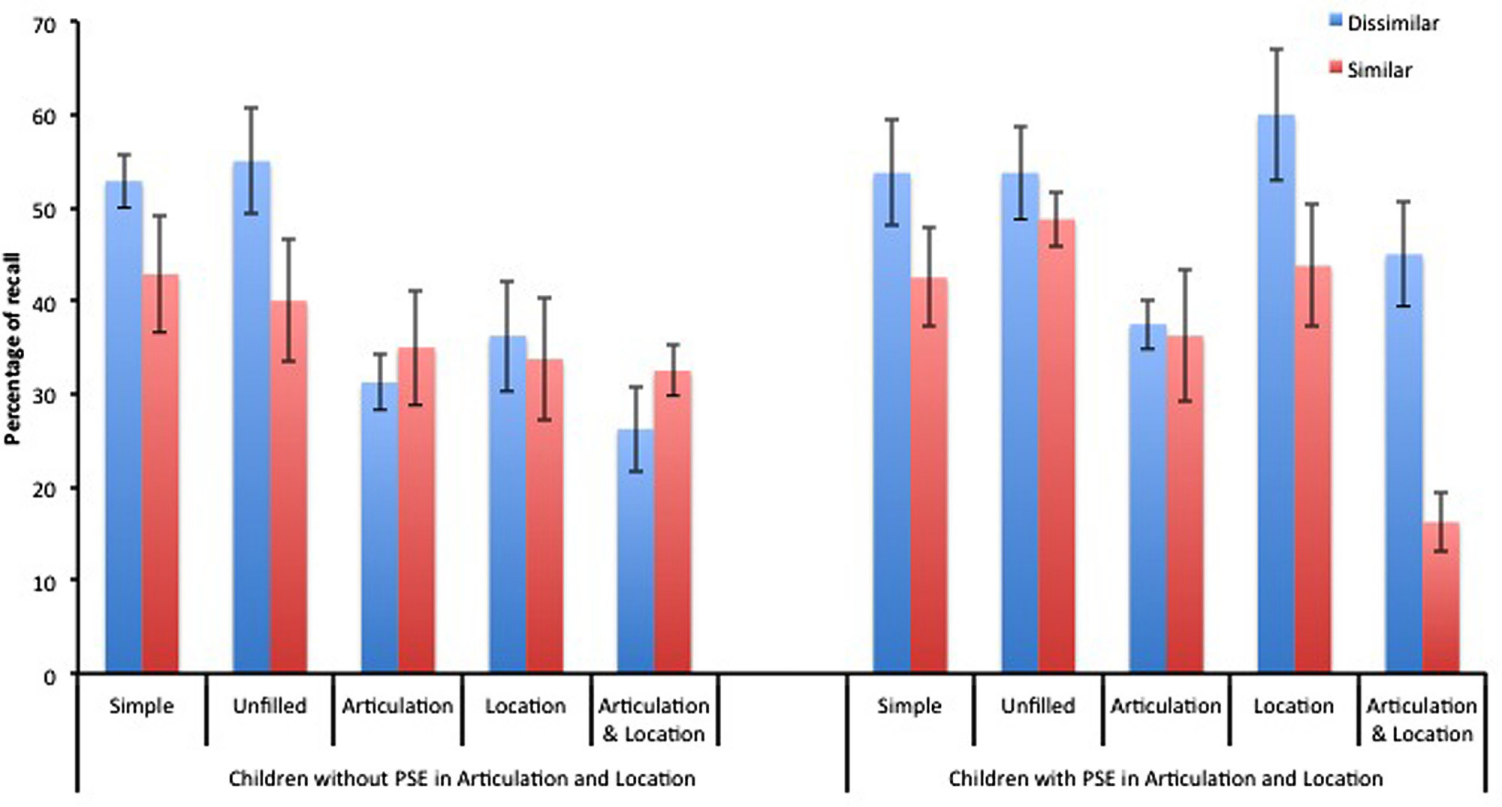
**Percentage of recall according to the type of lists (phonologically dissimilar or similar words), the occurrence of a concurrent articulation and the addition of a concurrent location judgment task for the two subgroups of children (without and with a phonological similarity effect, PSE)**. Y bars represented SE.

The examination of the children’s performance in the *Articulation and Location* condition led to suspect different patterns of behavior and to distinguish two subgroups. Indeed, recall performance of some children was clearly not affected by the phonological similarity of the memory words, whereas it was for other children. To explore this issue, we segregated our sample into two subgroups based on the absolute size of the PSE in the *Articulation and Location* condition. The difference in recall between the dissimilar and the similar lists was negative or null for eight children (mean difference = -6%) or it was positive and above the mean of the overall sample for eight other children (mean difference = 29%). Three children were not included in any group, because the size of their PSE was equal to sample mean. The former constituted the subgroups named children without PSE and the latter the group with PSE (cf. **Figure 3**). The size of these groups being so small, we avoid reporting any statistical analysis, but preferred to describe the pattern of results as it could give some insights about the maintenance mechanism used by children.

In the concurrent activities, the two groups produced a similar number of “*oui*” (7.4 and 7.5 for children without and with PSE, respectively), and achieved a comparable rate of accuracy (69 and 70%, respectively) in the location judgment task, but the children without PSE (509 ms) were slower than the children with PSE to judge a location (478 ms). In the *simple span* task, both groups had relatively similar performance and both exhibited a PSE (**Figure 3**). In the complex span tasks, the two groups have different pattern of results. The children without PSE showed an absence of the PSE in any condition that required to perform another activity (either articulation or location judgment task) concurrently to maintenance. In the *Articulation*,* Location*, and* Articulation and Location* conditions, recall performance was comparable for dissimilar and similar lists. It was also poorer compared to the other group. On the contrary, when the delay of retention was unfilled, recall was as good as in the other group, and showed a PSE. The children with PSE exhibited a different pattern. The PSE appeared in conditions in which children performed a concurrent location judgment task, i.e., *Location*, and* Articulation and Location* conditions, but it disappeared in absence of location judgment task (i.e., *Unfilled* and *Articulation* conditions).

## DISCUSSION

The present study was twofold. First, it aimed at exploring the relationships between rehearsal and refreshing in the maintenance of verbal information in children’s working memory. This was inspired by two recent models of working memory, the last version of the multi-component model (Baddeley, 2012) and of the TBRS model (Camos et al., 2009). Both models suggest the existence of two maintenance systems that could allow the maintenance of verbal information. Moreover, some studies in adults have already shown that these two mechanisms are independent. The second aim found its root in Tam et al.’s (2010) study in children and concerned how the phonological characteristics of the memory items would affect working memory maintenance.

After the first study by Tam et al. (2010), this experiment aimed at extending the examination of the relationships between rehearsal and refreshing in the maintenance of verbal information in children. The findings showed that 8-year-old children are able to use rehearsal and refreshing to maintain memory items, as it is expected from children of that age. Indeed, impeding each of the two mechanisms of maintenance resulted in a reduction of children’s recall performance. More interestingly, the manipulation of the availability of the two mechanisms by introducing a concurrent articulation, which impedes rehearsal, and a concurrent task, that reduces the use of refreshing, did not lead to interaction. Moreover, the Bayesian analysis supported the null hypothesis. This is interesting for two reasons. First, this is the first attempt in children to examine the relationships between the two mechanisms in a fully crossed design. Second, this result replicated in children what is known in adults. Thus, we can suspect that, from 8 to adulthood, rehearsal and refreshing are two independent mechanisms. To extend this work, it remains to understand if this independence between rehearsal and refreshing observed at 8 and present in young adults also characterizes the functioning of working memory in younger children. The age of emergence of rehearsal use was recently reassessed, and it was suggested that children younger than 7 could use this maintenance mechanism (Al-Namlah et al., 2006; Tam et al., 2010; Jarrold and Tam, 2011; Henry et al., 2012). Similarly, although refreshing is conceived to emerge at seven (Barrouillet et al., 2009; Camos and Barrouillet, 2011), attention may play a role in working memory before this age (Barrouillet et al., 2009; Tam et al., 2010; Bertrand and Camos, 2015). Within this scope, examining pre-schoolers becomes crucial to understand the development of working memory.

This study also allowed us to examine how the phonological similarity of the memoranda affects working memory maintenance. We replicated Tam et al.’s (2010) finding. In working memory tasks, lists of phonologically dissimilar words were better recalled than lists of similar words, as they were also in the simple span task. This result extended Tam et al.’s (2010) finding in Brown–Peterson task to another working memory paradigm, complex span task. However, and contrary to adults’ studies in which the PSE depends exclusively on the availability of rehearsal, variation in the PSE was far more complex in children.

More fine-grained analysis allowed us to distinguish two subgroups of children. For some children, the PSE emerged exclusively if no concurrent activity had to be performed, neither a concurrent task nor a concurrent articulation. In absence of concurrent activity, recall for dissimilar lists was better than for similar lists. Otherwise, recall did not differ across types of lists and was rather poor compared to other children performing the same complex span task. For other children, the emergence of the PSE seems depending on the occurrence of a concurrent attentional demanding task. Recall was poorer for similar (vs. dissimilar) lists when children performed a concurrent task, but no difference occurred in absence of competing attentional demand. How can we understand these two distinct patterns of recall? In accordance with the idea that the PSE is an index of the use of rehearsal, we can suggest an interpretation. However, we are aware that this idea was recently challenged (Jarrold and Citroën, 2013), and our interpretation should be better conceived as opening new perspectives of research than as a definite proposal. In previous work, we have shown that adults are able to adaptively choose one of the two maintenance mechanisms according to the constraints of the task. When they have to perform a concurrent attention-demanding task, they favor the use of rehearsal, because it is poorly attention demanding. On the contrary, if the maintenance through rehearsal could lead to some confusion, as it is the case for phonological similar lists, adults favor maintaining words through refreshing. We propose that the difference in children’s pattern of recall performance in complex span task resulted from difference in strategy choice. We excluded the idea that the different patterns reflect differences in other characteristics (like words knowledge, efficiency of rehearsal), because the two groups of children observed in the present study did not differ in simple span task in which recall depends on rehearsal. Our interpretation relies on the same idea as in our adults study, i.e., children can adapt their maintenance mechanism to some characteristics of the task. However, each group may have a systematic bias for one of the two systems.

Let’s consider the former group of children. We suggest that these children favor a language-based system. Thus, they would maintain information by subvocal rehearsal, which results to a PSE in *simple span* and *unfilled* condition. However, when they performed a concurrent articulation, their default system could not work, their recall drastically reduces and the PSE disappears. Moreover, when they have to perform the location judgment task, they would also rely on language to control their activity. The use of language to voluntary control cognitive activities has long been documented (e.g., Vygotsky, 1935/1986; Luria, 1969), and is especially apparent in task switching (e.g., Baddeley et al., 2001; Emerson and Miyake, 2003; Kray et al., 2008). By construction, complex span tasks require to switch between two tasks: the storage of memory items and the concurrent task. These children could use language to help them controlling the achievement of complex span tasks. However, this will be at the cost of inducing a concurrent articulation, which reduces recall and removes the PSE. A similar functioning could be suggested to account for the results observed in the other group of children, but with a systematic bias for the attentional system. In this case, things are easier to conceive. Children use attentional refreshing in absence of concurrent attentional task. When attention is distracted by a concurrent task, they back up to a non-attentional maintenance mechanism, rehearsal, which is sensitive to PSE. To summarize, considering that children have a systematic bias for one of the two systems and that they are able at 8 years of age to adapt their strategy choice could provide a sufficient account of the different patterns observed in the present study. However, we admit that this interpretation is speculative and based on a rather small amount of observations. Further work will have to confirm the existence of these two distinct patterns. Moreover, it should examined if these two groups of children differ on the basis of individual characteristics or if they represent two stages in development.

Finally, this study explored the relationships between rehearsal and refreshing. Rehearsal has been studied extensively in short-term and working memories. Recent debates appeared and challenged its role in accounting for development (Jarrold and Citroën, 2013; Jarrold and Hall, 2013), but its functioning and effects on recall is relatively well understood. On the contrary, refreshing was less studied, and remains a rather obscure mechanism. The exact functioning underlying refreshing of memory traces is still uncovered, and many suggestions have been put forward. Refreshing can be conceived as retrieval from long-term memory (Loaiza and McCabe, 2012), reconstruction of representations (Barrouillet and Camos, 2015), reactivation through attentional focusing (Johnson, 1992), or memory scanning (Cowan, 1992; Vergauwe and Cowan, 2015). It can also be conceived as consolidation or elaborative rehearsal. As suggesting by Jolicoeur and Dell’ Acqua (1998), consolidation transforms transient sensory inputs into a more durable form of memory. Thus, perceptual memories are strengthened, and these latter representations can maintain information over a longer time. By contrast, elaborative rehearsal is the enrichment of these short-term memory representations based on knowledge stored in long-term memory (McCabe, 2008). All these proposals aim at understanding how memory traces are maintained in the short term. Although they differed on the description of the mechanism, they all agree that this maintenance relies on central attentional resources, resources that have to be shared with concurrent cognitive task. The present study did not provide any hints for disentangling these different theoretical proposals. Throughout this paper, when referring to refreshing, it could stand for any of these processes, including consolidation and elaborative rehearsal. However, what the present study showed is that an attentional mechanism can sustain the maintenance of verbal information in children, and that this mechanism is distinct from subvocal rehearsal. Future research efforts should be made to reveal the nature of refreshing.

## CONCLUSION

To conclude, this study brings some evidence on the independence of subvocal rehearsal and attentional refreshing in the maintenance of verbal information in children. It also provides some examination on how the phonological characteristics of the memoranda affect recall performance in complex span tasks. Finally, it enlightens the complexity of the factors that support the choice of a maintenance strategy in children, and proposes some future directions of research to understand working memory and its development.

## ETHIC STATEMENT

The study was anonymous, conducted in accordance with the French ethical regulations, approved by the institutional review board of the Laboratoire d’Etude de l’Apprentissage et du Développement, and by the Académie de Bourgogne. Moreover, the caregivers of each participant gave written informed consent.

## Conflict of Interest Statement

The authors declare that the research was conducted in the absence of any commercial or financial relationships that could be construed as a potential conflict of interest.

## Footnotes

1 One child failed to perform the *Simple span* task, although she did all the complex span tasks. Her data were excluded for this analysis only.
